# Atmospheric Aerosol Assisted Pulsed Plasma Polymerization: An Environmentally Friendly Technique for Tunable Catechol-Bearing Thin Films

**DOI:** 10.3389/fchem.2019.00183

**Published:** 2019-04-02

**Authors:** Vincent Jalaber, Doriane Del Frari, Julien De Winter, Kahina Mehennaoui, Sébastien Planchon, Patrick Choquet, Christophe Detrembleur, Maryline Moreno-Couranjou

**Affiliations:** ^1^Department of Materials Research and Technology, Luxembourg Institute of Science and Technology, Belvaux, Luxembourg; ^2^Department of Chemistry, University of Mons, Mons, Belgium; ^3^Department of Environmental Research and Innovation, Luxembourg Institute of Science and Technology, Belvaux, Luxembourg; ^4^Center for Education and Research on Macromolecules, University of Liege, Liege, Belgium

**Keywords:** tunable catechol films, plasma polymerization, coating, surface modification, dry process

## Abstract

In this work, an atmospheric aerosol assisted pulsed plasma process is reported as an environmentally friendly technique for the preparation of tunable catechol-bearing thin films under solvent and catalyst free conditions. The approach relies on the direct injection of dopamine acrylamide dissolved in 2-hydroxyethylmethacrylate as comonomer into the plasma zone. By adjusting the pulsing of the electrical discharge, the reactive plasma process can be alternatively switch ON (t_ON_) and OFF (t_OFF_) during different periods of time, thus allowing a facile and fine tuning of the catechol density, morphology and deposition rate of the coating. An optimal t_ON_/t_OFF_ ratio is established, that permits maximizing the catechol content in the deposited film. Finally, a diagram, based on the average energy input into the process, is proposed allowing for easy custom synthesis of layers with specific chemical and physical properties, thus highlighting the utility of the developed dry plasma route.

## Introduction

Since the discovery of the versatile chemistry of polyphenols, and more particularly of catechols, an ever-growing number of bio-inspired catechol-based polymers have been reported and successfully exploited for the production of advanced materials, such as water-resistant or biomedical adhesives, multifunctional anti-corrosion, or anti-wear coatings and, more recently, of sustainable energy storage devices (Sedó et al., 2013; Wilker, 2014; Patil et al., 2018). These new functional materials rely on the unique and versatile chemical properties of catechols. Indeed, catechols are redox-active and/or chelating groups, or even spin-traps for radicals, to cite only few of them. Also, catechols can easily be oxidized into reactive quinones that can react with nucleophiles, such as amines or thiols, leading to strong irreversible covalent bond formation (Patil et al., 2018).

In the last years, tremendous efforts in the fields of polymer synthesis and thin film processing have been done in order to control the preparation and deposition of catechol-bearing polymer films. Wet chemistries, combined or not to catechol protection/deprotection reactions, have been by far the major explored routes. Recently, as an alternative to polydopamine (pDA) coatings (Lee et al., 2009), chemical methods based on the deposition of pre-formed polymers bearing catechols from aqueous or organic solutions, via the layer-by-layer (Faure et al., 2011) or nanogels techniques (Faure et al., 2012a,b,c) have rapidly emerged, offering an up-scalable character to catechol-based coatings. However, these techniques are most often requiring the use of catechol-bearing polymers produced by multi-step and wet chemical pathways.

Since decades, plasma polymerization processes are intensively explored as promising alternatives for the formation of organic coatings. Indeed, the plasma generates highly reactive electrons, ions, atoms, and radicals that initiate polymerizations of unsaturated as well as saturated monomers (Kobayashi et al., 1974; Friedrich, 2011) In particular, the so-called Atmospheric pressure Dielectric Barrier Discharge (AP-DBDs) can be considered as a sustainable process as it does not involve any catalyst or solvent, and works under ambient atmosphere. The product waste is considered to be negligible as the film formation requires solely the combined injection of monomers with a carrier gas, such as helium or argon. Importantly, AP-DBDs are also up-scalable, can be easily integrated into existing production lines (Massines et al., 2012), and can be configurated for the treatment of 2D and/or 3D substrates presenting complex geometries. A large diversity of polymer coatings characterized by exceptional properties (e.g., antifouling Pandiyaraj et al., 2018, superhydrophobic Levasseur et al., 2012, etc.) or functionalized with various chemical groups such as epoxy (Camporeale et al., 2015), anhydride (Manakhov et al., 2012; Permyakova et al., 2018), carboxylic (Chen et al., 2018; Cools et al., 2018) or amine (Borris et al., 2007), can be produced by this process.

We recently reported the first plasma deposition of reactive catechol functionalized thin films by exploiting a liquid-assisted DBD approach (Mauchauffé et al., 2016a,b). The method relies on spraying a liquid mixture, composed of dopamine-acrylamide (DOA) dissolved in a comonomer (vinyltrimethoxysilane - VTMOS) onto a substrate, and subsequently proceeding to its plasma polymerization. The catechol and quinone bearing-coatings were successfully exploited for the elaboration of antibiotic-degrading surfaces and dual antibacterial and antibiofouling coatings (Moreno-Couranjou et al., 2018). Based on a similar approach, Wang et al. reported the preparation of two-dimensional patterned coating surfaces by dipping the substrate into a dopamine solution and polymerizing it by a plasma torch (Wang et al., 2017). Despite promising results, both approaches might suffer from non-wetting phenomena relying on the nature of the liquid monomer mixture and the substrate surface free energy, thus leading to inhomogeneous coatings. Therefore, the development of a novel dry and substrate-independent method that would enable the fast and facile preparation of homogeneous catechol-bearing polymer thin films with tunable catechol contents is still a challenge.

In this work, we report the first atmospheric pulsed plasma process that allows the one-step deposition of thin films presenting a tunable catechol content, morphology and thickness from a catechol-bearing vinyl monomer, dopamine acrylamide (DOA), combined to 2-hydroxyethyl methacrylate (HEMA) as a comonomer. HEMA was selected considering its convenient chemical reactivity towards DOA (Sasikala et al., 2015) and for the poly(HEMA) properties such as biocompatibility, non-toxicity, and non-antigenicity (Folkman and Moscona, 1978). The liquid mixture of monomers nebulized under an argon flow is injected into the plasma discharge zone where it is polymerized. The pulsing of the discharge appears as a key parameter to tune the layer morphology as well as the chemical functionality of the coating. This novel atmospheric pressure plasma process is therefore a promising sustainable technique for the direct, facile and scalable synthesis of tunable catechol-bearing thin films under solvent-free conditions.

## Experimental

### Materials

Plasma depositions were carried out on mirror polished stainless steel (SS) disks (304-8ND, APERAM, 2 cm diameter, 0.8 mm thickness) and two face-polished silicon wafers (Sil'tronix), notably for IR and AFM analyses, respectively. A home synthesized N-(3,4-dihydroxyphenethyl)acrylamide, called dopamine acrylamide (DOA) was produced according the procedure reported in Patil et al. (2015). The 2-hydroxyethyl methacrylate monomer (HEMA; 97%) was purchased from Sigma–Aldrich and used as received or after a purification step using a silica column. The Micro-BCA protein assay kit was purchased from Thermo Scientific. The liquid precursor mixture was obtained by dissolving solid DOA in HEMA during 30 min at a final 3.3 mg mL^−1^ concentration. SS substrates were cleaned by successive ultrasonic washings in acetone (5 min), and absolute ethanol (5 min) and further dried under a nitrogen flux. Prior deposition, SS substrates were activated through an Ar: O_2_ (19 standard liter per minute (slm):1slm) plasma treatment in continuous mode at 1.6 W cm^−2^ during 2 min. This plasma treatment ensures an ultimate cleaning and promotes the anchoring of the deposited functional layer.

### Atmospheric Plasma Deposition

Coatings were deposited using an atmospheric pressure dielectric barrier discharge (AP-DBD) process. The discharge was produced between two plane-parallel high voltage electrodes covered by alumina and a moving table as the ground electrode, thus generating a ~19 cm^2^ plasma discharge area. Samples were placed on the moving table and the electrode gap adjusted at 1 mm. The precursor solution was nebulized using a OneNeb injector system (Agilent) and a syringe pump to control its flow delivery (5 μL min^−1^). The droplet size distribution of the precursor spray, examined using the Spraytech laser diffraction system from Malvern, was centered around 1 μm. During the deposition process, the plasma was generated using a Corona generator (SOFTAL electronic GmbH) with a 10 kHz sinusoidal signal. Coatings were deposited using a modulated electrical excitation, i.e., pulsed wave with different t_ON_ and t_OFF_ pulses. The total process gas flow was fixed at 20 slm of argon (99.999 %, Air Liquide). The applied voltage was measured with an HV probe (Lecroy, PPE, 20 kV, 100 MHz). The current was estimated using a Lecroy (CP030, 50 MHz) Hall Effect current probe. The charge was determined by measuring the voltage drop across a 9.4 nF capacitor connected in series with the ground electrode. Data were recorded with a digital oscilloscope (Lecroy, WS 42XS, 300 MHz bandwidth). The average power (W_a_) values were calculated according to Equation (1).

(1) $$\begin{array}{r} W_{a}\;\left(W\right)=\;W_{Peak}\;\left(W\right)\times \;\frac{t_{ON}}{t_{ON}+\;t_{OFF}} \end{array}$$

Optical Emission Spectroscopy (OES) measurements have been carried out using an ARC SpectraPro-2500i spectrometer equipped with a PIXIS 400 CCD detector. Process conditions were a peak power of 30 W and a pulsed discharge at 1:400 ms in pure argon.

### Film Characterizations

The mass of the films was estimated by weighing the substrate before and after deposition using a Sartorius ME-36S microbalance. Each deposition condition was carried out, at least, on 4 substrates. As a result, the reported value corresponds to the average values. The thickness of the layer was estimated using a contact profilometer (P-17, KLA Tencor). The growth rates in weight and thickness were determined considering the measured masses, thicknesses and the effective deposition duration.

The coating morphologies were observed by Scanning Electronics Microscopy (SEM) using a Hitachi SU70 HRSEM instrument. Prior observations, in order to avoid charging effects, the samples were coated with a 7 nm platinum layer using a BAL-TEC MED020 metallizer equipment.

Atomic force microscopy (AFM) analyses were carried out in constant amplitude tapping mode (2.8V) with scanning speeds ranging from 1 Hz to 2. The tip (HQ15NSC15) came from Micromasch (325 KHz, 40 N/m). Images were reprocessed with the SPIP software. Roughness parameters were calculated by subtracting the mean plane.

UV-visible absorption spectroscopy analyses were carried out using a PerkinElmer Lambda 950 UV–vis-NIR (InGaAs) spectrophotometer equipped with an integrating sphere. Samples were dissolved in a 2 mL absolute ethanol solution.

Fourier Transform Infrared Spectroscopy (FT-IR) analyses were performed in the Attenuated Total Reflectance (ATR) mode on a Hyperion 2000 spectrometer (Bruker). The spectra, acquired at 32 scans and a 4 cm^−1^ spectral resolution, were processed using the Opus 7.5 software.

X-ray Photoelectron Spectroscopy (XPS) analysis was carried out using the Kratos AXIS Ultra DLD instrument, equipped with a hemispherical energy analyzer and a monochromatic X-ray source (Al Ka, hν = 1486.6eV) operating at 150 W. Global and high-resolution XPS spectra were recorded with a pass energy of 160 and 40 eV, respectively. Spectra were reprocessed with the Casa XPS software. All spectra were calibrated using the aliphatic C1s carbon peak at 285 eV. The C 1s core level was fitted with 5 main components: (i) C-C/C-H (285 eV), (ii) C-CO-O (285.7 eV), (iii) C-O / catechol (286.7 eV), (iv) C = O/O-C-O (288 eV), and (v) O-C = O (289 ± 0.1 eV) bonds (Beamson and Briggs, 1992). The full width at half maximum (FWHM) and the Gaussian/Lorentzian ratio were fixed at 1.0 ± 0.1eV and 30%, respectively. The experimental uncertainty related to the XPS surface elemental composition is around 2 at %.

Mass spectrometry analysis was carried in order to evaluate the structure of the plasma deposited layers. Electrospray ionization mass spectrum was recorded using Waters Synapt G2-Si mass spectrometer. Firstly, the coating was dissolved in an absolute ethanol (2 mg mL^−1^). The solution was then diluted 10 times in methanol and 4 μL of a NaI solution (4 mg mL^−1^ in acetonitrile) was added. This solution was then directly infused in the ESI source with a typical flow rate of 5 μL min^−1^ using a capillary voltage of 3.1 kV, a source temperature of 100°C and a desolvation temperature of 200°C. The quadrupole was set to pass ions from m/z 100 to m/z 2,000. All ions were transmitted into the pusher region of the time-of-flight analyzer (Resolution~20,000) for mass-analysis with 1 s integration time. Data were acquired in continuum mode until acceptable average data were obtained (<5 min).

### Quantitative Estimation of the Catechol Content in the Deposited Thin Films

To estimate the catechol content in the deposited thin films, two different methods were exploited, both relying on the catechol oxydo-reduction property. The first technique is based on a commercial Micro BCA kit, originally exploited for protein quantification and later extended to catechol (Liu et al., 2015). Briefly, the method relies on the reduction of Cu^2+^ to Cu^+^ by catechol. The Cu^+^ then complexes with bicinchoninic acid (BCA) which absorbs light at 562 nm (Slocum and Deupree, 1991). The second technique, issued from own developments, is based on the combination of AgNO_3_ immersion tests with potentiometric measurements. Indeed, immersed in the silver salt solution (Ag^+^), catechol-bearing thin films operate as a reducing agent, thus converting silver ion into silver nanoparticle (Ag^0^) (Lee et al., 2007, 2008). The silver salt reacting with catechol group in a 1:1 molar ratio (Equation 2), the amount of Ag^+^ consumed during the reaction should equal the initial amount of catechol groups. As an indirect measurement, the estimation of the Ag^+^ concentration in the AgNO_3_ solution, before and after the layer immersion step, indicates the catechol amount in the deposited films.

(2) $$\begin{array}{r} Ag^{+}+\;catechol\;\to \;Ag^{0}+quinone \end{array}$$

In the dark, SS coated with plasma layers of known thicknesses were immersed in a 6 mL of an AgNO_3_ solution (1mg mL^−1^) during 24 h and under stirring. Afterwards, the solutions were recovered and titrated by potentiometric measurements. A known volume of a NaCl solution (5.339 10^−4^ M) was added while the evolution of the potential difference, between a silver electrode and an E_Ag_/_AgCl_ reference electrode, was followed. Hence, by graphical reading, it was possible to deduce the equivalent volume, V_eq_, allowing to determine the final Ag^+^ concentration as reported in Equation (3). Preliminary measurements carried out on selected plasma polymerized (pp) layers confirm the reliability and reproducibility of the method. Indeed, Ag^+^ consumptions of 1 and 26 % (compared to the initial Ag^+^ concentration) are measured for pure pp(HEMA) (i.e., layer of reference) and pp (DOA-HEMA), respectively.

(3) $$\begin{array}{l} C_{\text{Ag+}(\text{unknown})}\;\times V_{\text{eq}(\text{experimental value})}=\\ \;C_{\text{Cl-}(\text{known})}\times V_{\text{Cl-}(\text{known})}\; \end{array}$$

### Cytoviva® Dark Field Hyperspectral Imaging for Silver Nanoparticles Visualization

In the dark, SS coated with plasma layers were immersed in a 6 mL of an AgNO_3_ solution (1 mg mL^−1^) during 24 h and under stirring. Afterward, the solution was collected and a drop placed on microscope glass slide. Visualization was performed using Cytoviva® hyperspectral imaging system (Cytoviva Inc., Auburn, Alabama, USA) mounted on Olympus BX-43 optical microscope. Images were captured at 60-fold magnification with oil immersion using hyperspectral camera controlled by environment for visualization ENVI software (version 4.8 from Harris Corporation, Melbourne, FL, USA and modified by CytoViva®, Inc.).

## Results and Discussions

### Atmospheric Plasma

**Figure 1:**
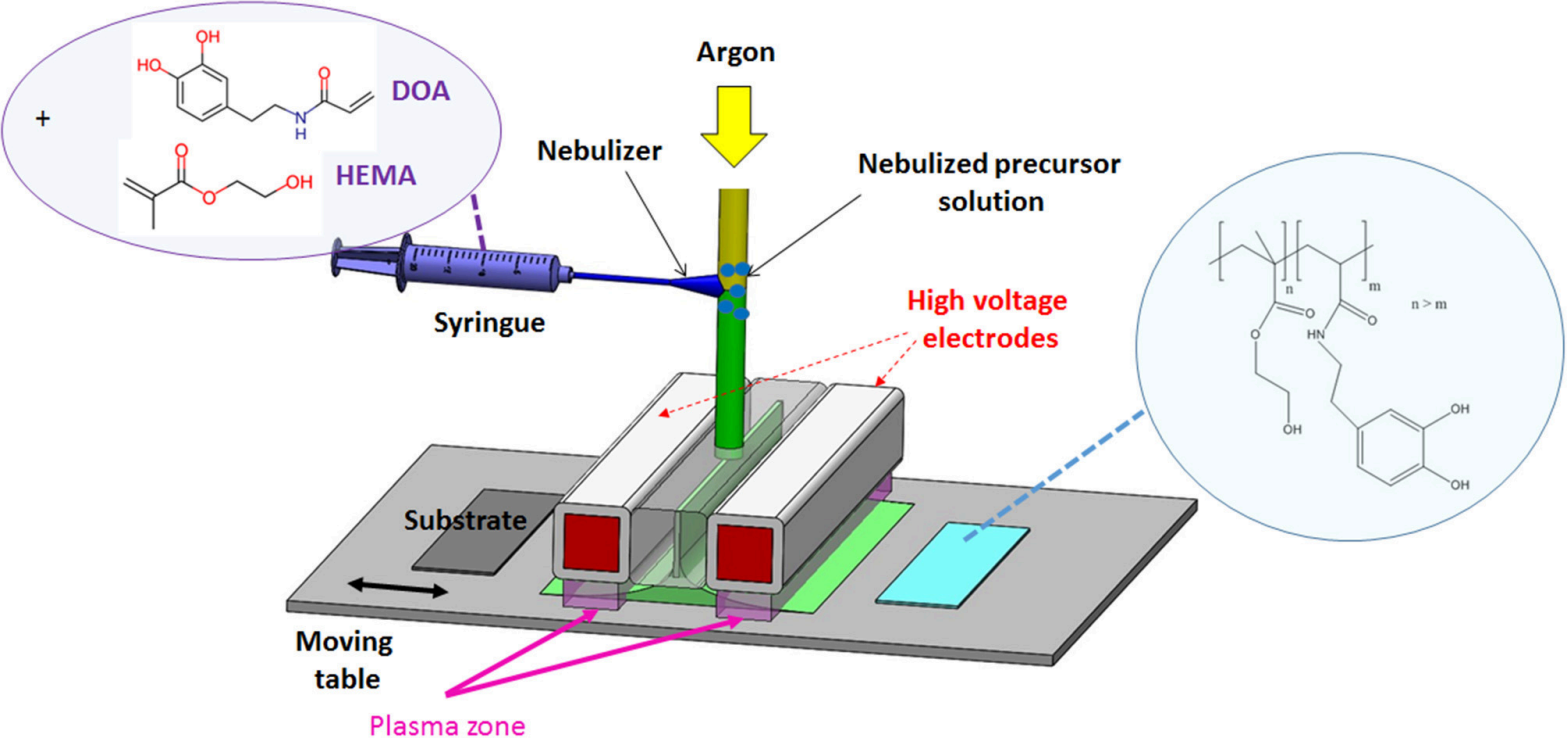
Schematic representation of the atmospheric aerosol assisted plasma process.

Figure 1 illustrates the open-air atmospheric aerosol-assisted Dielectric Barrier Discharge process operating with argon. A mixture of DOA and HEMA (DOA:HEMA = 0.3:99.7 wt/wt) was nebulized and injected in the plasma zone. OES of the argon dielectric barrier discharge was used to identify various chemical species in the plasma gas phase. In the 300–400 nm region, an intense spectral line from •OH at 309 nm and small emission lines due to NH and N_2_ at 338 and 359 nm, respectively were identified (Fang et al., 2016). The •OH emission peak was probably caused by fragmentation of H_2_O molecules, which diffused from ambient air. Similarly, the detected nitrogen species might originate from the diffusion of ambient air into the discharge. Finally, in the 700–800 nm range, atomic oxygen emission at 777 nm and different emission lines attributed to argon were also observed. For a fixed 1.6 W cm^−2^ power density (Figures S1, S2), the influence of the pulsing of the plasma discharge (and thus on different switching ON and OFF periods) on the deposition rate, chemistry and morphology of the films is investigated.

### Influence of Different Plasma Switching ON Durations

**Figure 2:**
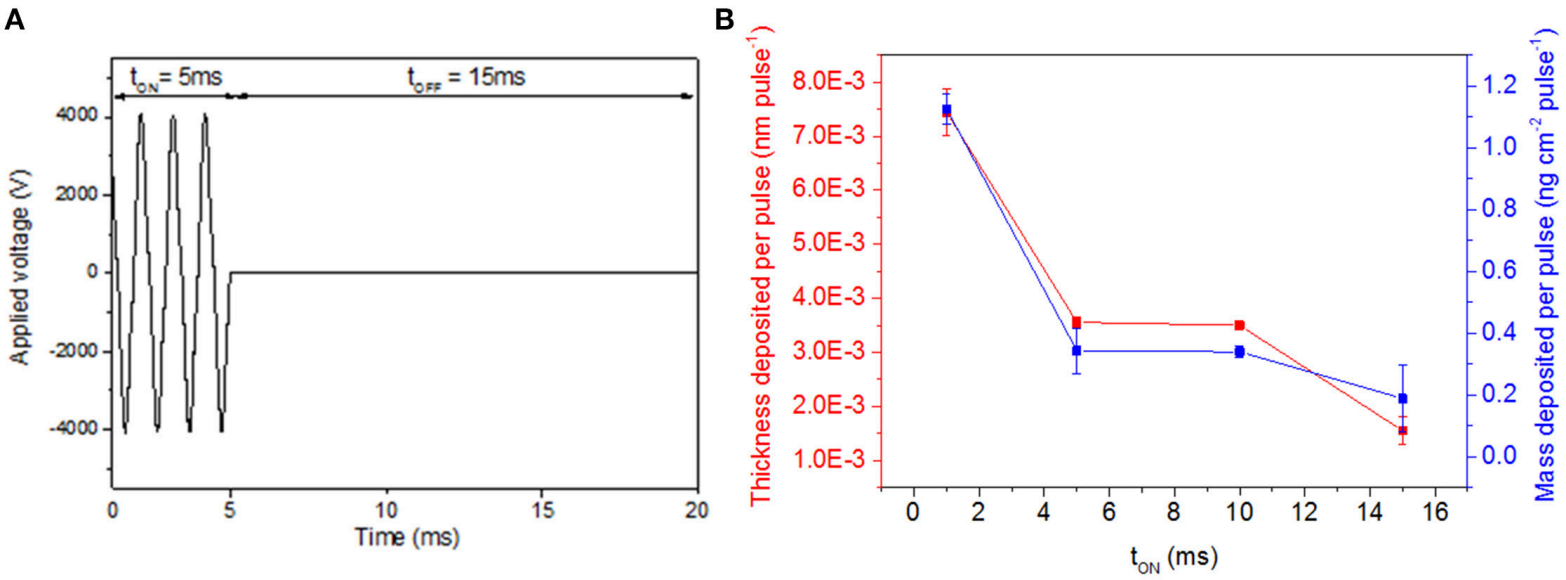
**(A)** Example of pulsed voltage with t_ON_ of 5 ms and t_OFF_ of 15 ms **(B)** mass deposited per pulse (i.e., t_ON_ + t_OFF_) for a 15 ms t_OFF_ and t_ON_ values ranging from 1 to 15 ms (error bars: means ± SD, *n* = 3).

In a first set of experiment, t_ON_ is varied from 1 to 15 ms while t_OFF_ is fixed at 15 ms to ensure the renewing of the gas mixture between the electrodes during each ON-time. Figure 2A reports an example of pulse (i.e., t_ON_ + t_OFF_) composed of a 5 t_ON_ and 15 ms t_OFF_. Figure 2B displays the evolution of the layer thickness and mass deposited per pulse as a function of different t_ON_. For a 1 ms t_ON_, the mass deposited is 1.13 ng cm^−2^ with a layer thickness of 7.4 10^−3^ nm per pulse. Increasing t_ON_ induces a depletion in the mass deposition per pulse with the value that decreases to 0.2 ng cm^−2^ pulse^−1^ with a t_ON_ of 15 ms. This result suggests that part of the monomer dissociates into volatile fragments for higher t_ON_, consequently it does not contribute to the film growth. Interestingly, considering the dynamic character of the deposition technique, the thickness of the coating can be easily adjusted by programing the number of runs (Figure S3).

**Figure 3:**
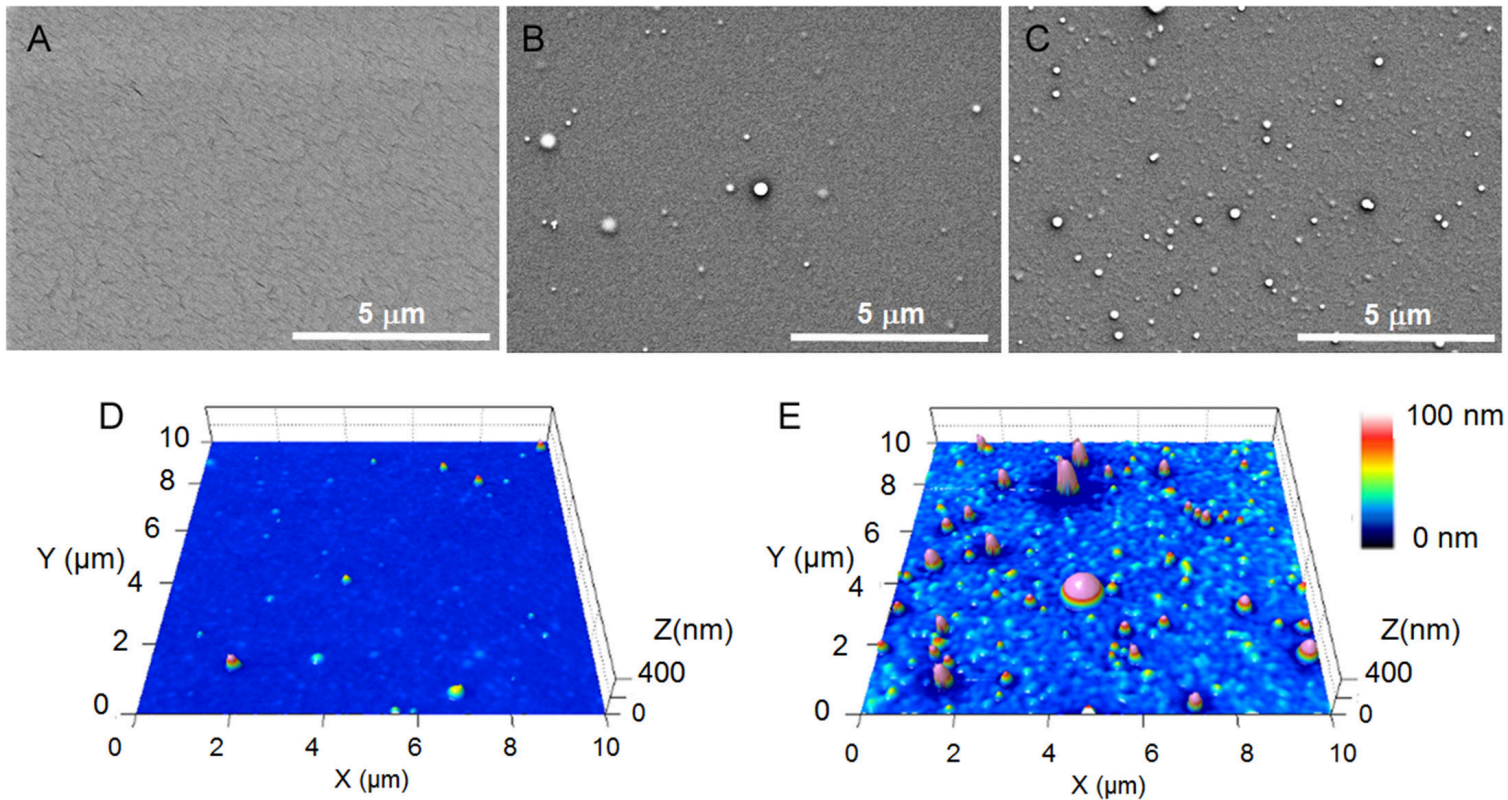
SEM pictures for coatings deposited at 15 ms t_OFF_ and t_ON_ of: **(A)** 1 ms, **(B)** 5 ms, and **(C)** 10 ms. AFM pictures for coatings deposited at 15 ms t_OFF_ and t_ON_ of: **(D)** 5 ms and **(E)** 10 ms.

According to SEM and AFM pictures (Figure 3) of 100 nm thick coatings, it appears that pulsing of the discharge is likely to affect both deposition rate and film morphology. Indeed, a short t_ON_ duration (i.e., 1 ms, Figure 3A) provides a smooth and pinhole-free coating that is covering homogeneously the entire substrate surface. By increasing t_ON_ from 5 to 10 ms, rough coatings are formed, with the roughness that is increasing with t_ON_ (0.9 nm vs. 10.3 nm). Numerous particles are also observed that present an average diameter size up to 267 nm for a 10 ms t_ON_ (Figure 3E).

**Figure 4:**
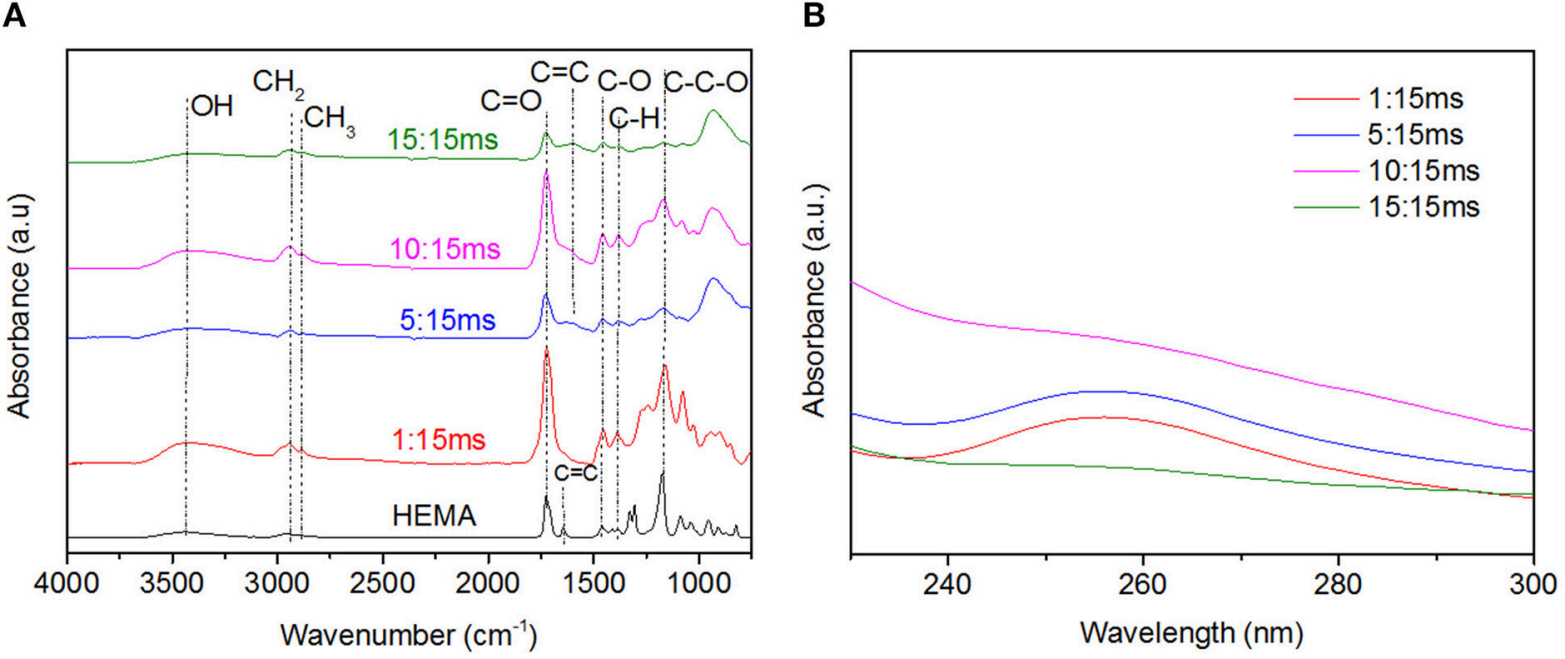
pp(DOA-HEMA) layers deposited at 15 ms t_OFF_ and t_ON_ ranging from 1 to 15 ms. **(A)** Normalized FT-IR spectra, HEMA is given as reference, **(B)** UV spectra.

**Figure 5:**
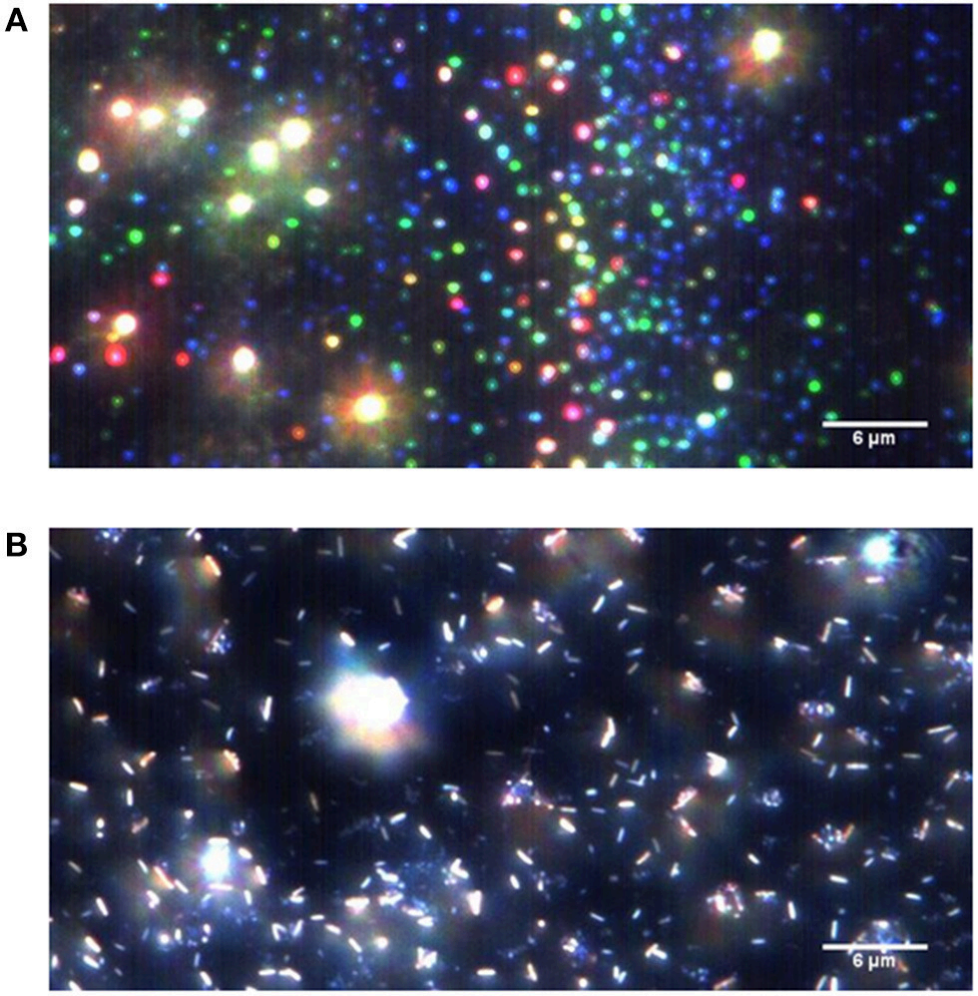
Dark field microscopy images of solutions resulting from pplayers immersed during 24 h in an AgNO_3_ solution at 1 mg ml^−1^ for pplayer deposited at 1:15 ms **(A)** and 10:15 ms **(B)**.

To gain insight into the coating chemistry, FT-IR measurements are carried out. Figure 4A presents the IR spectra of pure HEMA (as reference) and coatings deposited at different t_ON_. Irrespective of the deposition conditions, one can notice that the film spectra present some HEMA units fingerprint peaks, such as C-O bending in CH_2_-O at 1,454 cm^−1^, C-C-O stretching at 1,166 cm^−1^ and C-O in primary alcohol at 1,079 and 1,031 cm^−1^ stretching. The disappearance of the HEMA C=CH_2_ peak at 3,107 cm^−1^ along with the shift toward higher wavenumber of the C=O peak (from 1,718 cm^−1^ to 1,726 cm^−1^) suggests an efficient polymerization reaction through the C=C bonds, associated to the loss of conjugation for the C=O bond. In contrast, any peak relative to the DOA contribution was noticeable. One explanation may be the initial low DOA content in the precursor mixture. Interestingly, films deposited at high t_ON_ present: (i) a band broadening around 1,273 cm^−1^, usually related to strong monomer fragmentation processes and resulting in high crosslinked films (Veuillet et al., 2017) and (ii) a new peak contribution at 1,623 cm^−1^, probably related to the formation of C=C bonds. Complementary bulk UV-Vis analyses are carried out in order to detect the presence of catechol group in the films deposited at different deposition conditions. Preliminary assays reveal an absorbance at 300 nm only detected in a DOA-HEMA solution and related to catechol (Figure S4). Such result is consistent with the absence of absorbing group in plasma polymerized (pp) layer from (HEMA), noted pp(HEMA), thus confirming the relevance of this analysis. Figure 4B reports the UV spectra of different pp(DOA-HEMA) layers (presenting an averaged 75 nm thickness) in the 200–300 nm region. Interestingly, a maximal of absorbance at 260 nm, attributed to catechols, is observed for films deposited at 1 and 5 ms t_ON_. The variation in the maximal wavenumber position might be attributed to different catechol environments, the film being dissolved in ethanol prior UV measurements (Figure S5A). Indeed, plasma deposition carried on polished mirror stainless-stell disks and analyzed in UV reflectance mode indicate a catechol peak at 300 nm (Figure S5B). Considering the layer deposited at 10 ms and 15 ms t_ON_, a small shoulder is only noticeable for 10 ms, suggesting a lower catechol content. To support the presence of catechol in the film, complementary Cytoviva (i.e., Enhanced darkfield Hyperspectral microscope) analyses have been carried out. This technique enables rapid characterization of nanoparticles and is mainly employed by biologists to investigate histological samples (Vallotton et al., 2015; Mehennaoui et al., 2018). In this work, the technique was exploited to track silver nanoparticles issued from the reduction of silver salt by the catechol. Indeed, it is now well-established that silver nanoparticles (AgNPs) are efficient at absorbing and scattering light and have a color that depends upon the size of the particle (Théoret and Wilkinsona, 2017). Here, a pp(layer) deposited at 1:15 ms was immersed in AgNO_3_ solution overnight. The solution was collected and analyzed via Cytoviva. The dark field microscopy image, reported in Figure 5A, highlights the presence of numerous bright blue and green points. As shown in Figure S6A, the bright blue color, due to a surface plasmon resonance (SPR), is peaked at a 450 nm wavenumber and might be correlated to the presence of AgNPs with a 60 nm size. In Figure S6B, the maximal intensity of the spectral profile for the green points is around 540 nm. Such profile shift to higher wavelengths is related to an increased diameter of the AgNPs, estimated around 100 nm. Similarly, a pp(DOA-HEMA) film deposited at 10:15 ms was reacted with AgNO_3_. In Figure 5B reporting the dark field microscopy picture, no bright point is visible, indicating the absence of AgNPs. The presence of rods might be related to some film fragments issued from the film. Hence, in accordance with the reported UV results (i.e., Figure 4B), it can be concluded that using a 10 ms t_ON_ induces the degradation of the catechol-based monomer, explaining its absence in the deposited film. These results are in agreement with the IR ones, suggesting that depositions carried out at high t_ON_ values lead to coatings with poor monomer structure retention.

**Figure 6:**
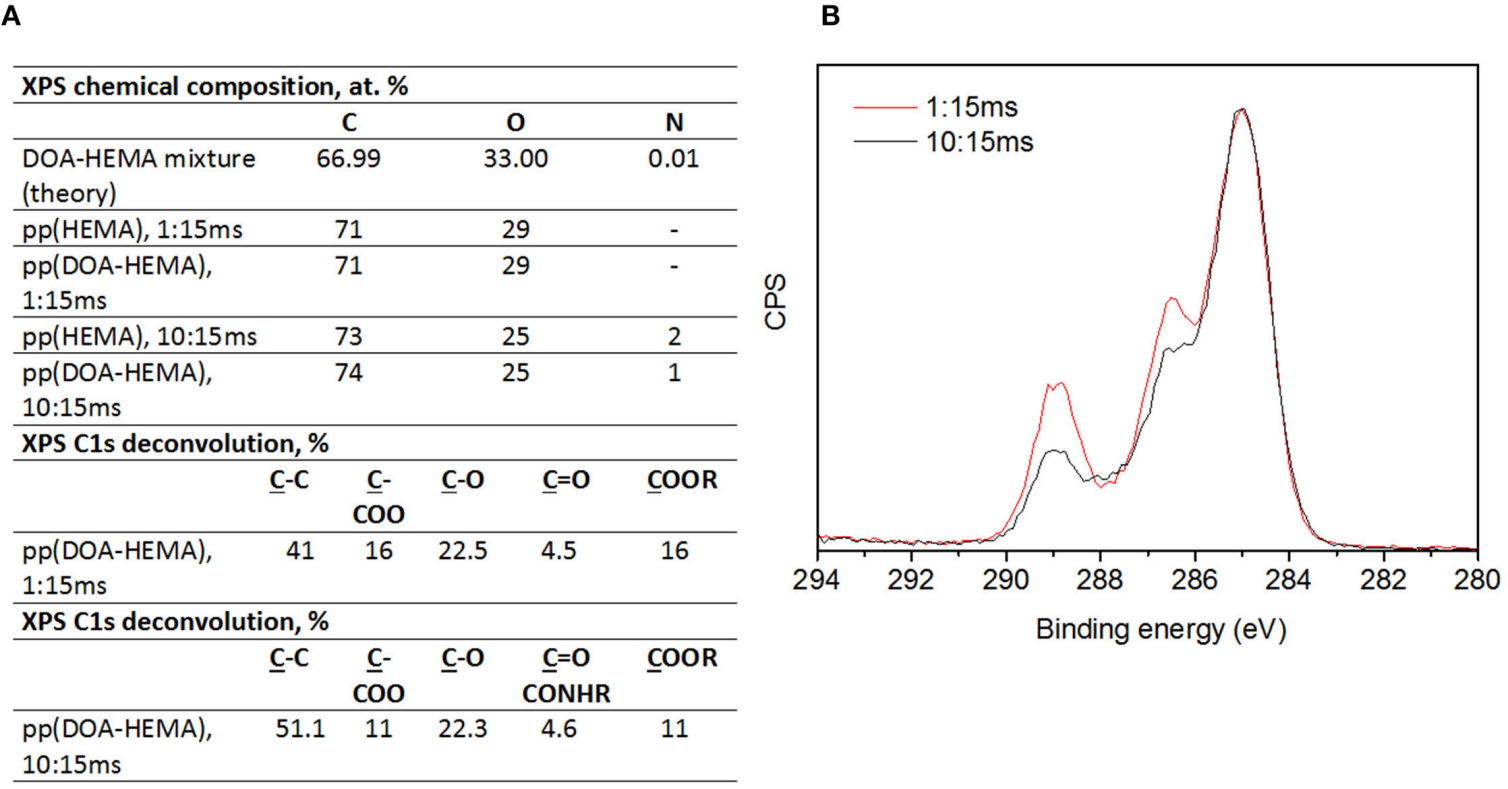
Table summarizing XPS analyses for pp(HEMA) and pp(DOA-HEMA) deposited at 1:15 ms and 10:15 ms **(A)**, XPS C1s envelope overlaps of pp(DOA-HEMA) deposited at 1 and 15ms t_ON_ and 15 ms t_OFF_**(B)**.

Figure 6 summarizes the results of the XPS analyses of pplayers from pure HEMA and DOA-HEMA deposited at 1 and 10 ms t_ON_. Irrespective of the precursor nature, coatings deposited at 1 ms t_ON_ present a similar C:O content around 71:29 at.% (Figure 5A). Nitrogen is not detected, suggesting neither air contamination in the deposited film considering pp(HEMA) nor DOA detection in pp(DOA-HEMA). This latter observation may be explained by the extremely low DOA content in the monomer mixture. Indeed, nitrogen is present in the precursor mixture at 0.01 at. %. Pp(HEMA) and pp(DOA-HEMA) deposited at 10 t_ON_ present similar C and O amounts as well as a N content around 1–2 at.%. Here, the presence of nitrogen in pp(HEMA) coating might be related the influence of air contamination due to predominant gas phase reactions in such operating condition. From the XPS C1s overlappings of pp(DOA-HEMA) layers deposited at 1:15 ms and 10:15 ms (Figure 5B), it appears that the t_ON_ value strongly influences the carbon environments. Indeed, the C1s deconvolution of layers deposited at short t_ON_ (Figure 5A) present higher ester contribution (16 vs. 11 %), which is consistent with IR results. It is worth noting that both deposition conditions contribute also to the HEMA fragmentation as both layers contain a novel contribution attributed to C=O bond.

To quantitatively estimate the catechol content in the deposited film, a micro-BCA assay kit, already applied to pDA coatings (Shin et al., 2011), was firstly exploited. However, a lack of specificity toward catechol compound was observed, with catechol detection for native SS substrates and pp(HEMA). Therefore, a method relying on the oxidation of catechols with an excess of silver nitrate, followed by potentiometric titration of excess silver cation is carried out before and after being in contact with plasma deposited layers. This technique allows quantifying the catechol content in pp(layers) (see experimental section for details) with an accuracy of 1%. This analytical method is applied to two pp(DOA-HEMA) deposited at different t_ON_. The catechol densities are estimated to be 34 and 18 μg cm^−2^ for the pplayer deposited at 1 and 5 ms t_ON_, respectively. This result is fully consistent with the previous bulk and surface layer characterizations, highlighting a lower monomer structure retention when working at higher t_ON_ duration. Indeed, using pulsed discharges with long t_ON_ duration ensure plasma depositions according to a so-called monomer deficient regime. During t_ON_, high energy is transferred to monomer molecules. The collision rate between monomers and electrons/ions is increased, leading to an increased monomer dissociation rate and a loss of the precursor structure in the deposited film (Kakaroglou et al., 2015). Gas phase reactions dominate the film growth, thus favoring particles/agglomerates formation and rough-to-powder-like coatings.

In the next section, the lowest t_ON_ value (i.e., 1 ms) is selected for its low monomer fragmentation impact. The study is extended to explore the effects of varying the t_OFF_ in an attempt to determine the optimal t_ON_/t_OFF_ ratio to maximize the functional group retention for catechol-bearing plasma polymers.

### Influence of Different Plasma Switching OFF Durations

**Figure 7:**
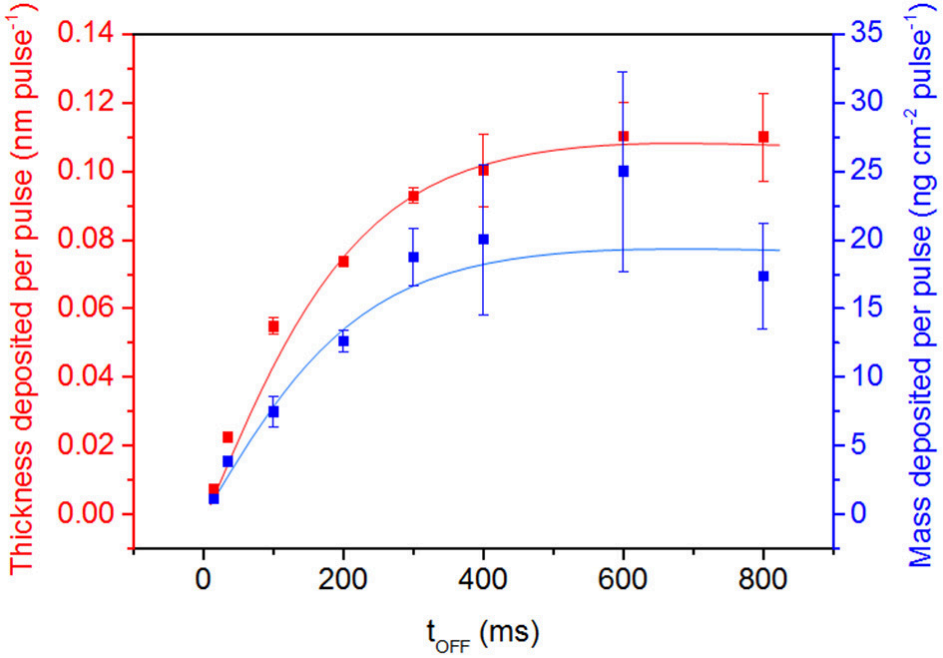
Evolution of the layer thickness and mass deposited per pulse for different t_OFF_ durations and a fixed 1ms t_ON_ (error bars: means ± SD, *n* = 3).

Figure 7 displays the layer thickness and mass deposited per pulse according to different t_OFF_ duration. Interestingly, the thickness and mass rates exhibit a similar trend, characterized by a logarithmic layer growth for cycle values ranging from 15 to 400 ms and followed by a plateau. Therefore, it appears that the layer growth is the highest for a t_OFF_ of 400 ms.

**Figure 8:**
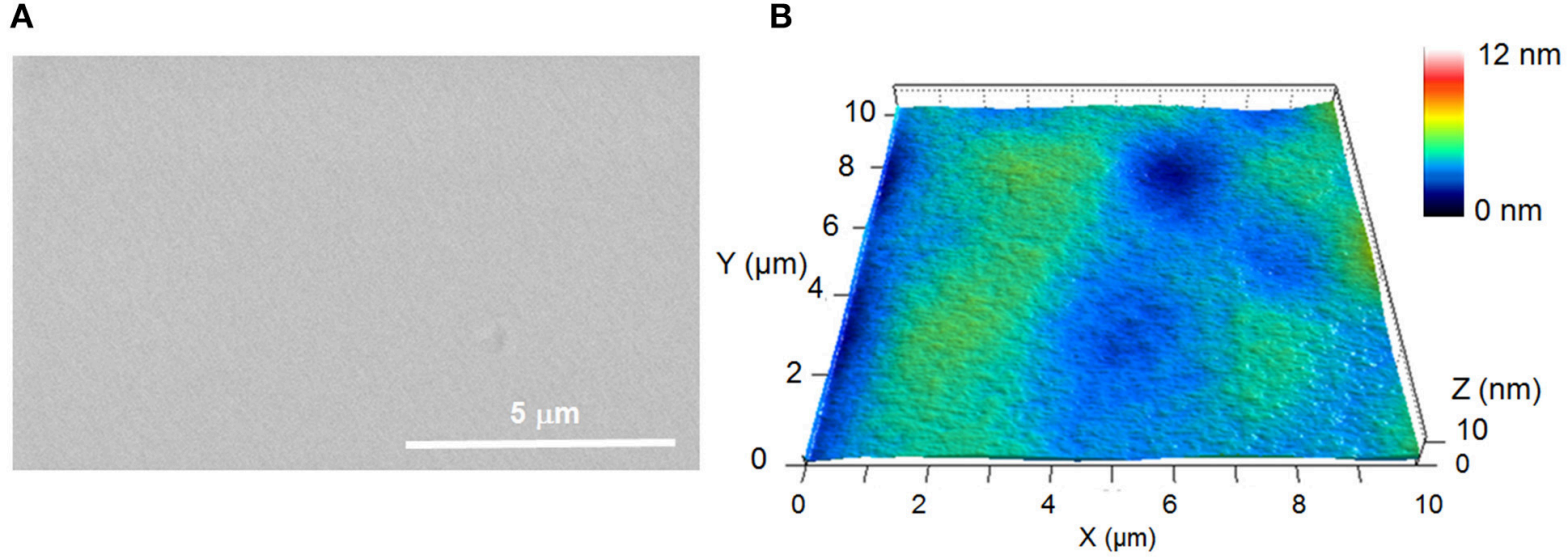
SEM **(A)** and AFM **(B)** pictures of pp(DOA-HEMA) deposited at 1:400 ms.

SEM and AFM analyses demonstrate that, irrespective of the depositions conditions, smooth and pinhole-free coatings covering homogenously the substrate are achieved with a maximal average roughness (Sa) of 1.5 nm. As an example, Figure 8 presents the SEM and AFM pictures of a 150 nm layer deposited at 1:400 ms.

**Figure 9:**
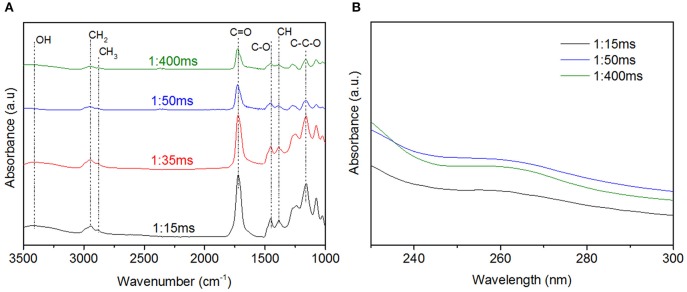
pp(DOA-HEMA) deposited at 1 ms t_ON_ and different t_OFF_ duration: Normalized FT-IR spectra **(A)** and UV analyses **(B)**.

Also, IR spectra of the deposited films are similar in all cases (Figure 9A). The absence of the monomer vinyl bands at 3,107 cm^−1^ and 1,638 cm^−1^ confirms an efficient polymerization via carbon double bonds C=C bonds. The series of peaks located at 1,454 cm^−1^ (C-O), 1,166 cm^−1^ (C-C-O) and 1,079 and 1,031 cm^−1^ (C-O in primary alcohol) are in line with a well-retained poly(HEMA) structure in the deposited layers. Complementary UV analyses (Figure 9B) definitely confirm the DOA incorporation in all deposited layers as the result of the catechol peak absorbance at 264 nm.

**Figure 10:**
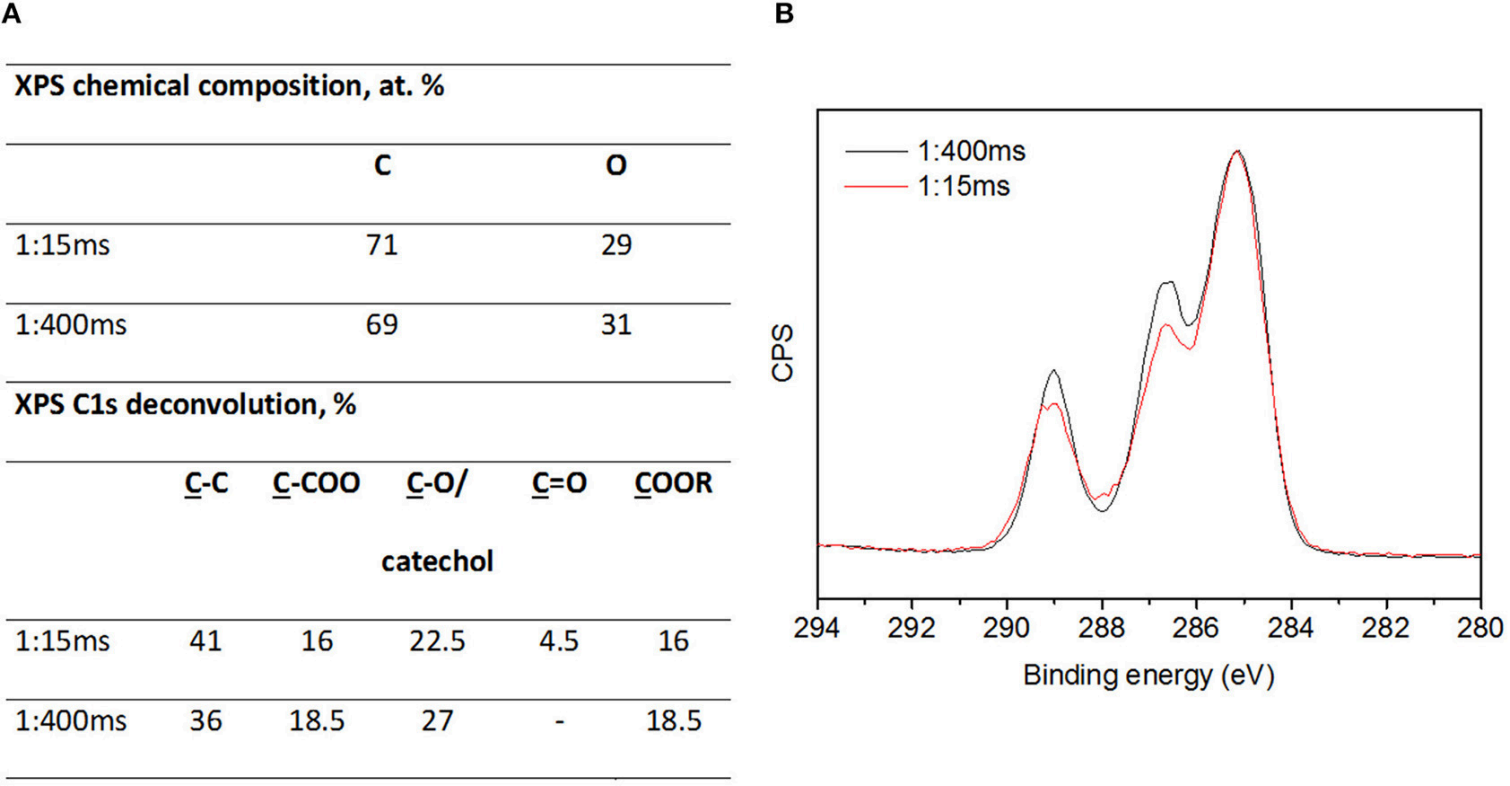
XPS analysis of pp(DOA-HEMA) deposited at 1:15 ms and 1:400 ms **(A)**. XPS C1s envelope overlaps of pp(DOA-HEMA) deposited at 1:15 ms (black curve) and 1:400 ms (red curve) **(B)**.

According to Figure 10 reporting the XPS analysis of pp(DOA-HEMA) deposited at 15 and 400 ms t_OFF_, it appears that the coatings present a similar chemical composition but different carbon environments. Indeed, increasing t_OFF_ from 15 to 400 ms considerably improves the monomers structure retention in the layers, namely ester (COOR) and catechol (C-O/catechol) from HEMA and DOA, respectively, and limits the formation of secondary groups, such as C=O groups resulting from HEMA fragmentation.

The catechol content in films deposited at 1:50 ms and 1:400 ms is then estimated by the potentiometric titration as described above. Extending t_OFF_ from 50 to 400 ms significantly increases the catechol content from 51 to 138 μg cm^−2^. These results are fully consistent with the previous measurements, mentioning a 33 μg cm^−2^ catechol density for a 1:50 ms deposited layer. Optimal depositions are therefore achieved with a 1:400 ms pulsed discharge leading to smooth and catechol rich layers.

**Figure 11:**
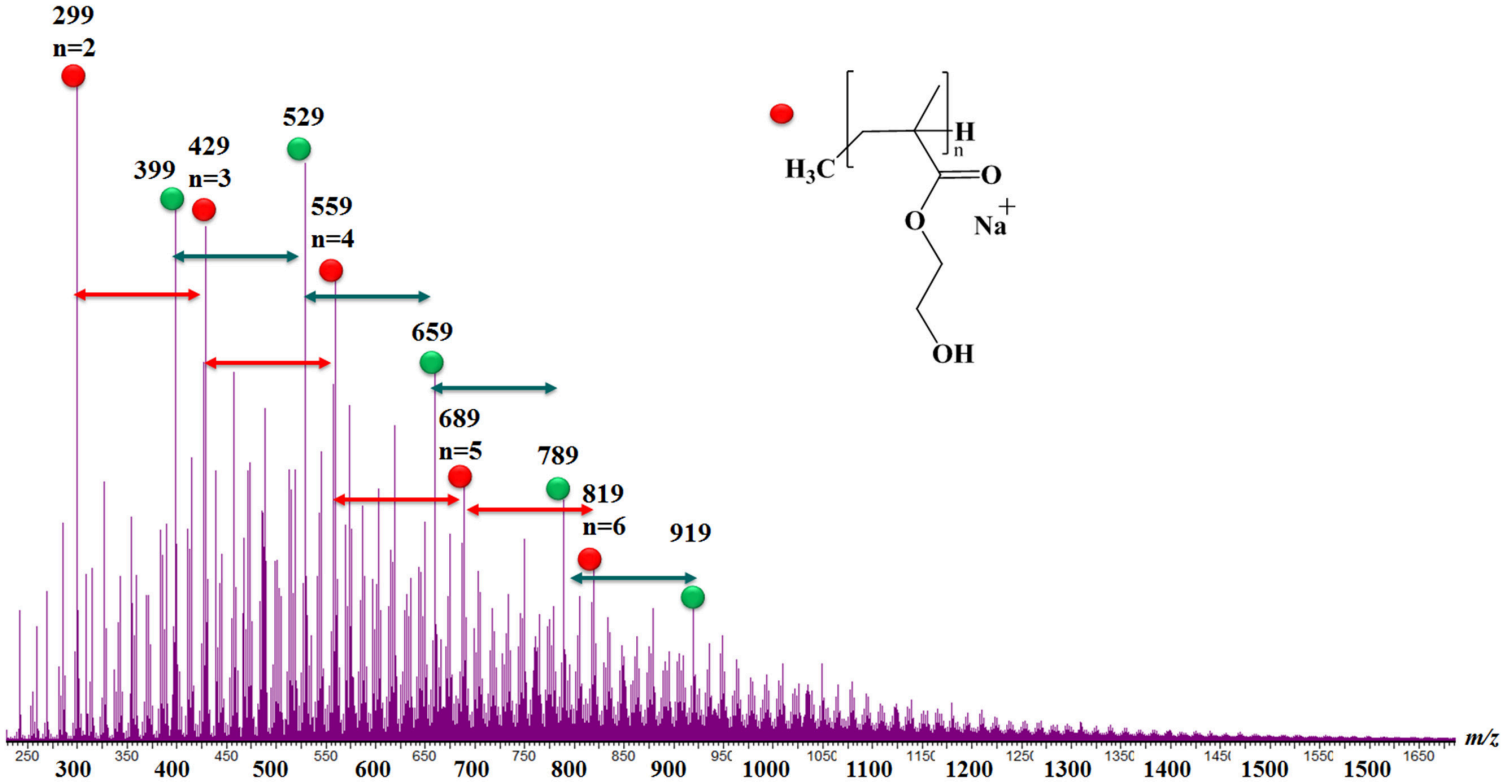
ESI mass spectrum recorded for a pp(DOA-HEMA) deposited in a 1:400 ms pulsed mode.

To gain insight into the plasma deposited layer (pplayer) structure and composition, complementary mass spectrometry analyses are carried out (Jackson and Simonsick, 1997). In this purpose, Electrospray Ionization (ESI) Mass spectrometry analyses are performed by using a direct injection of the diluted pplayer solution. The as-obtained mass spectrum is reported in Figure 11. Interestingly, various distributions of oligomer ions separated by 130 mass units are unambiguously identified. This mass difference is perfectly consistent with HEMA monomer mass unit, thus confirming a layer formation from radical polymerization reactions occurring during t_OFF_. In addition, MS analyses clearly highlighted many different end-groups (Figure S7). This result might be explained by the numerous and various chemical species issued from the monomer fragmentation generated during t_ON_. Hence, in Figure 11, red dots distribution corresponds to poly(HEMA) with, formally CH_4_ as end-groups, and characterized by DP superior or equaled to 18. Considering the green dot distribution, this latter may be attributed to poly(HEMA) with one formaldehyde molecule loss. Many signals separated by 2u lower are also detected and might be attributed to H_2_ losses. Finally, by comparing pp(HEMA) and pp(DOA-HEMA) MS spectra, no significant modification of the mass spectrum is observed. This result might be explained by the fact that (i) only the soluble part of the polymer is analyzed by ESI-MS, (ii) the poor DOA concentration in the precursor mixture along with the huge number of different oligomer species present in the 250–1,500 mass range. Hence, using pulsed discharges favoring long t_OFF_ vs. short t_ON_ durations ensure plasma depositions according to a so-called monomer sufficient regime. This latter is defined by a concentration of active species involved in plasma polymerization lower than the monomer molecules density in the plasma zone, the layer growth being directly governed by the modulation of the electrical discharge. Hence, during each short t_ON_ (i.e., 1 ms), there is monomer activation and fragmentation as well as the generation of reactive sites at the substrate surface via UV irradiation, ion and electron bombardments. During the subsequent t_OFF_, so in the absence of any UV, ion or electron induced damage, conventional and heterogeneous free-radical polymerization reactions occur between radicals at the surface and fresh monomer molecules continuously injected and being absorbed at the surface (Ward et al., 2003). Such mild deposition conditions allow the formation of smooth layers with extremely high levels of structural retention of the precursors, here, catechol as well as ester functional groups.

**Figure 12:**
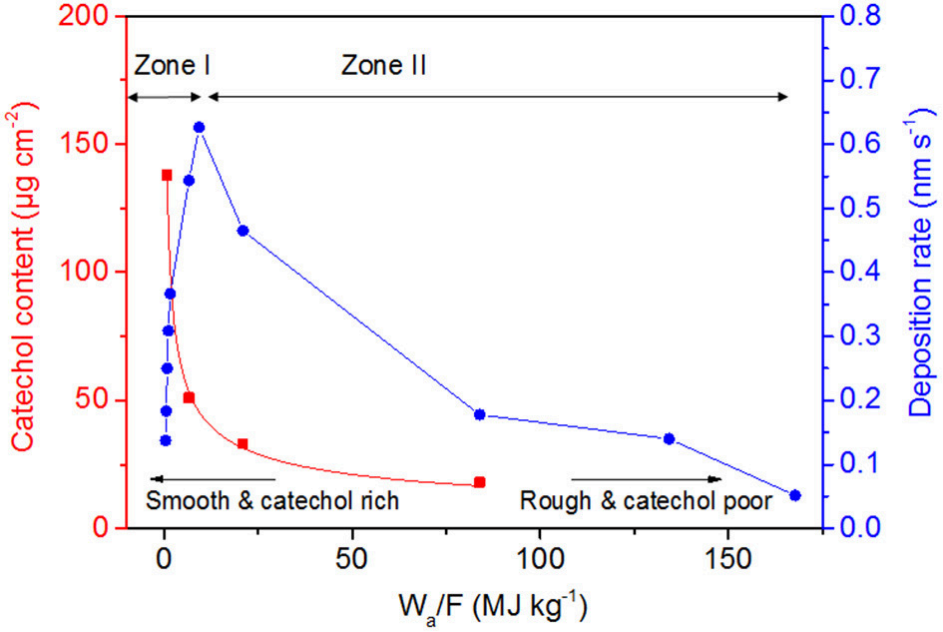
Evolution of the film catechol content, deposition rates and morphological trends as a function of W_a_/F. Zone I is defined by a 1 ms t_ON_ and t_OFF_ ranging from 15 to 800 ms. Zone II is defined by a 15 ms t_OFF_ and t_ON_ ranging from 1 to 15 ms.

Finally, as an attempt to correlate deposition kinetics and physico-chemical properties of deposited films, the evolution of the catechol content and deposition rates are plotted as a function of a W_a_/F factor (W_a_ and F corresponding to the average power in W estimated according to Equation 1 and the monomer flow rate fixed here at 8.9 10^−8^ Kg s^−1^ (i.e., 5 μL min-1), respectively) (Figure 12). Indeed, originally introduced for low-pressure plasma processes, this factor has been transposed to AP-DBDs (Hegemann et al., 2007, 2009; Nisol et al., 2016). According to Figure 12, two distinct deposition regimes are identified with a competition zone identified around 9.3 MJ/Kg corresponding to an optimal deposition rate of 0.62 nm/s. In zone I (i.e., monomer sufficient regime), defined by low values of W_a_/F (in the 0.4–9.3 MJ/Kg range), the deposition rate is increasing from 0.14 to 0.62 nm/s. In zone II (i.e., monomer deficient regime), defined by W_a_/F value exceeding 9.3 MJ/kg, the deposition rate decreases down to 0.05 nm/s. Figure 12 is therefore a useful and simple tool to select the appropriate conditions to prepare specific layers with targeted thickness, catechol content and morphology. Importantly, the experimenter should respect the t_ONand_ t_OFF_ values fixed in each zone as well as the precursor flow rate. Indeed, using similar W_a_/F values do not imply the formation of similar coatings (Kakaroglou et al., 2015). Hence, as an example, in <2.5 min, 50 nm thick and smooth coatings containing 100 ug cm^−2^ catechol can be synthesized by depositing at a W_a_/F of 1.7 MJ/kg (i.e., 1 ms t_ON_ and 200 ms t_OFF_). Similarly, 50 nm thick and rough coating containing 25 ug cm^−2^ catechol can be deposited in 8 min. using a W_a_/F of 50 MJ/kg (i.e., 3 ms t_ON_ and 15 ms t_OFF_).

## Conclusion

In summary, an atmospheric pressure pulsed plasma process, fed with a catechol-containing aerosol mixture, is proposed as an upscaling-friendly dry process for the facile and fast preparation of tunable catechol-bearing thin polymer films. By a judicious adjustment of the different plasma ON and OFF times, we have shown that the morphology, deposition rate and catechol content of the layers can be finely tuned. When short plasma discharge (t_ON_ of 1 ms) was associated to long t_OFF_ periods (400 ms), the deposition rate is high (0.25 nm s^−1^), homogeneous and catechol-rich films are formed, and low fragmentation of the monomers/polymer is noted. During t_OFF_ period, the monomer gas mixture is renewed between the electrodes, ensuring its polymerization from the species generated during t_ON_ time. In addition, a diagram has been set-up, thus facilitating the selection of deposition conditions to achieve layers with targeted specific thickness, morphology and catechol content. The low energy required to rapidly build films (0.07 W for 30 nm in 2 min) under atmospheric conditions, the solvent-free procedure and the absence of waste render this process highly attractive for the sustainable construction of functional coatings. Finally, due to the low-temperature involved in the plasma deposition process, the treatment of heat sensitive substrates, such as paper or polymer foils (Hilt et al., 2016), can be easily carried out.

## Author Contributions

All authors listed have made a substantial, direct and intellectual contribution to the work, and approved it for publication.

### Conflict of Interest Statement

The authors declare that the research was conducted in the absence of any commercial or financial relationships that could be construed as a potential conflict of interest.

## Supplementary Material

Figure S1 Applied voltage and discharge current measured in the AP-DBD operating in continuous mode and fed with a 5 μL min^-1^ precursor mixture.

Figure S2 Lissajous figure for the AP-DBD operating in continuous (CW) mode and fed with pure argon (black curve) and a 5 μL min^-1^ precursor mixture diluted (red curve).

Figure S3 Evolution of the thickness of pp(DOA-HEMA) films deposited at 1:15 ms according to the number of runs.

Figure S4 UV spectra of DOA in solution in HEMA at 3.3 mg mL^-1^.

Figure S5 UV spectra of pp(DOA-HEM) film deposited in a 1:400 ms pulsed mode on a silicon wafer and dissolved in ethanol (i.e, UV transmission mode) **(A)** and on stainless steel disks (i.e., UV reflectance mode) **(B)**.

Figure S6 Dark field microscopy image with the corresponding spectral profile for a blue point **(A)** and a green point **(B)** for a pp(layer) deposited at 1:15 ms.

Figure S7 End-groups composition estimated from ESI mass spectrum recorded for a pp(DOA-HEMA) deposited in a 1:400 ms pulsed mode.
