# Supplementary figures and images for: Atmospheric Aerosol Assisted Pulsed Plasma Polymerization: An Environmentally Friendly Technique for Tunable Catechol-Bearing Thin Films
Source: Front Chem. 2019 Apr 2;7:183. doi: 10.3389/fchem.2019.00183 (PMC6454202; doi:10.3389/fchem.2019.00183)

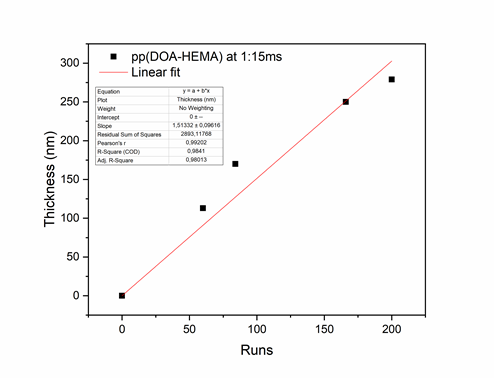

Supplement: Figure S3 — Evolution of the thickness of pp(DOA-HEMA) films deposited at 1:15 ms according to the number of runs. [file Image_3.TIF]

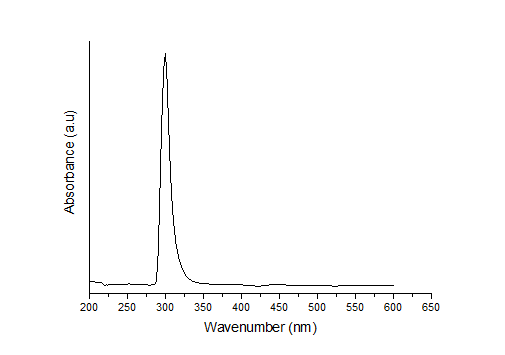

Supplement: Figure S4 — UV spectra of DOA in solution in HEMA at 3.3 mg mL-1. [file Image_4.TIF]

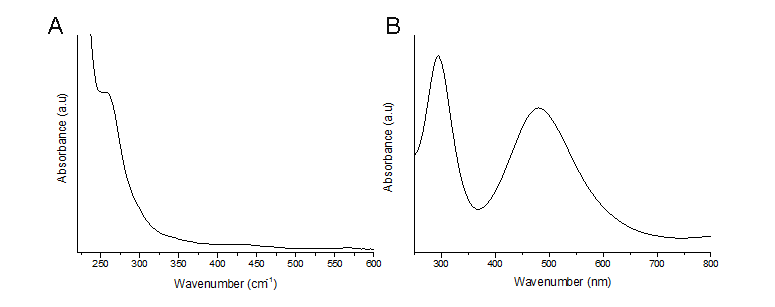

Supplement: Figure S5 — UV spectra of pp(DOA-HEM) film deposited in a 1:400 ms pulsed mode on a silicon wafer and dissolved in ethanol (i.e, UV transmission mode) (A) and on stainless steel disks (i.e., UV reflectance mode) (B). [file Image_5.tif]

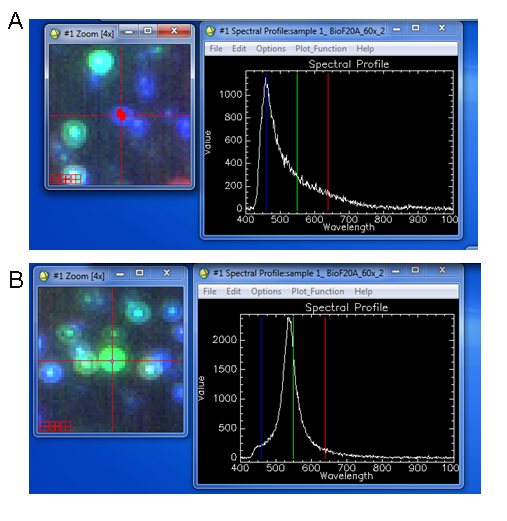

Supplement: Figure S6 — Dark field microscopy image with the corresponding spectral profile for a blue point (A) and a green point (B) for a pp(layer) deposited at 1:15 ms. [file Image_6.TIF]

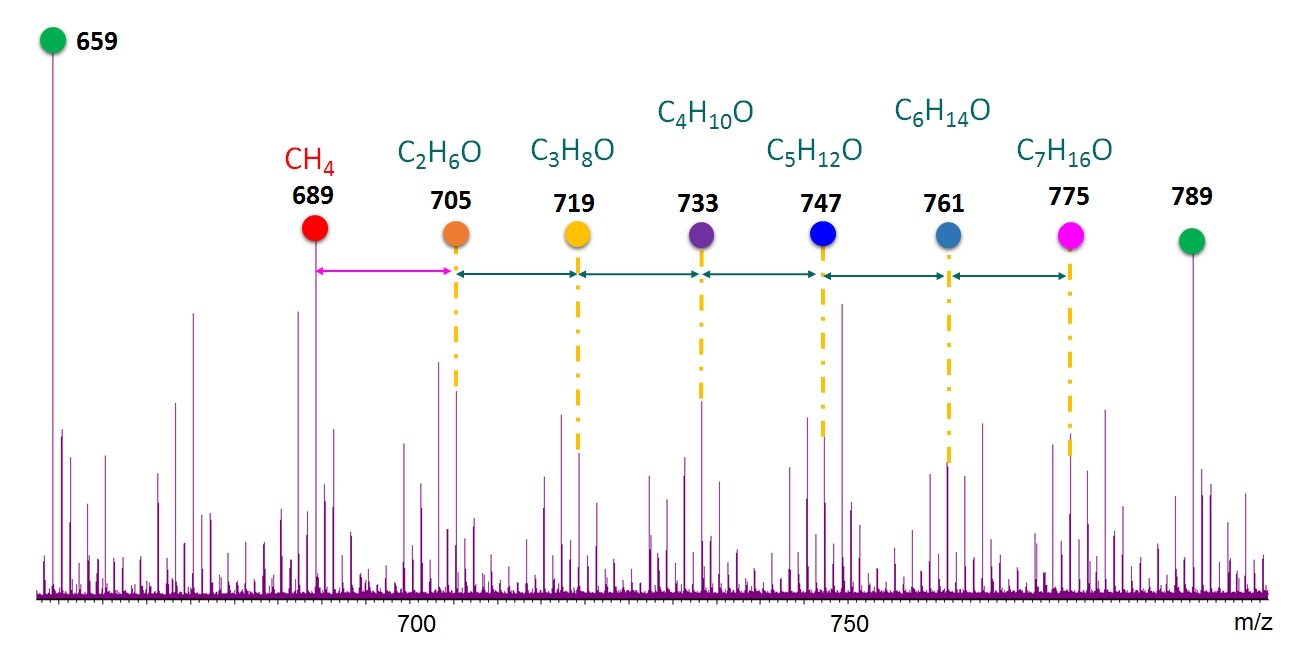

Supplement: Figure S7 — End-groups composition estimated from ESI mass spectrum recorded for a pp(DOA-HEMA) deposited in a 1:400 ms pulsed mode. [file Image_7.TIF]
